# A phase II study of concurrent chemo-radiotherapy with weekly nedaplatin in advanced squamous cell carcinoma of the uterine cervix

**DOI:** 10.1186/1748-717X-9-55

**Published:** 2014-02-18

**Authors:** Guangwen Yuan, Lingying Wu, Manni Huang, Nan Li, Jusheng An

**Affiliations:** 1 Department of Gynecologic Oncology, Cancer Hospital of Peking Union Medical College and Chinese Academy of Medical Science, No 17 Panjiayuan South Street, Chaoyang District, Beijing 00021, People’s Republic of China

**Keywords:** Nedaplatin, Concurrent chemo-radiotherapy, Advanced cervical carcinoma

## Abstract

**Background:**

To evaluate the efficacy and safety of concurrent chemo-radiotherapy with weekly nedaplatin for the treatment of advanced squamous cell carcinoma of the uterine cervix.

**Methods:**

Patients with stage Ib2 to IIIb squamous cell carcinoma of the uterine cervix were treated with concurrent radiotherapy and chemotherapy. The radiotherapy regimen included external beam radiation therapy (45–50.4Gy/25-28 fractions with central shielding after 30.6Gy) and high-dose-rate brachytherapy irradiation (35-49Gy/5-7 fractions to point A). The chemotherapy regimen was weekly intravenous infusion of nedaplatin (30 mg/m^2^, once weekly, 180 mg/m^2^ for 6 weeks).

**Results:**

Thirty patients were enrolled in this study from April 2010 to October 2010. The median age was 50.5 years (34–62). Three patients were at the clinical stage IIa2, twenty at stage IIb and seven at stage IIIb. Acute hematological toxicities included grade 3 leukopenia (8), neutropenia (5), anemia (2), grade 4 anemia (1), and grade 2 thrombocytopenia (6). Acute non-hematological toxicities included grade 2 liver disorders (1), diarrhea (2), nausea (2), and renal toxicity (1). There were not grade 3 or worse toxicities. 24 patients completed the treatment regimen and were evaluated for efficacy. 23 patients (95.8%) had CR (complete response) and 1 (4.2%) had PR (partial response) (100% response rate). The median follow-up duration was 36 months (23–39), during which three patients relapsed after the treatment. The 3-year PFS and OS rates were 87.5% and 91.7%, respectively.

**Conclusions:**

This phase II study suggested that concurrent chemo-radiotherapy with weekly nedaplatin was effective and safe for the treatment of advanced squamous cell carcinoma of the uterine cervix.

## Background

Although uterine cervical carcinomas have been treated via surgery, radiation therapy, or a combination of the two for a long time, the treatment outcomes were poor. In the 1990s, numerous attempts were made to improve the prognosis of advanced uterine cervical carcinoma with concurrent radiation therapy and chemotherapy. In February 1999, the US National Cancer Institute (NCI) stated that five randomized controlled trials of concurrent radiotherapy and chemotherapy (especially with the use of cisplatin) demonstrated that the treatment was effective to advanced uterine cervical carcinoma and decreased the risk of death by 30-50% [1-5]. The concurrent chemo-radiotherapy (CCRT) thus became the standard treatment of locally advanced uterine cervical carcinoma recommended by NCI and FIGO.

However, the adverse events (especially gastrointestinal events) associated with cisplatinare usually severe. The radiation therapy leads to gastrointestinal adverse reactions as well. The concurrent application of these two therapies significantly aggravates gastrointestinal adverse reactions, making it more difficult for patients to tolerate. Therefore, a drug with mild side effect is urgently needed for the concurrent chemo-radiotherapy.

Nedaplatin is an antineoplastic drug containing a platinum complex. It has better antitumor effects than cisplatin and less adverse reactions such as renal and gastrointestinal toxicities. A phase I clinical trial of nedaplatin showed that the drug should be administered by intravenous infusion with 100 mg/m^2^ at an interval of four weeks [6].

A phase II clinical trial using the dosage of 100 mg/m^2^ every four weeks showed a response rate of 46.3% (19/41 patients) in patients with uterine cervical carcinmoa [7], which was higher than that of cisplatin (35.9%, 14/39 patients). Although it demonstrated that nedaplatin had less severe nephrotoxicity than cisplatin, grade 3 or 4 myelosuppression happened in some patients (thrombocytopenia in 33.6% and leukopenia in 31.3%). The author suggested that the use of nedaplatin requires extreme caution [7].

Nedaplatin has a higher response rate in uterine cervical carcinoma than cisplatin and causes less gastrointestinal and renal side effects, and less fluid volume is needed. Nedaplatin is expected to provide a longer survival and better quality of life than cisplatin.

Two other phase I clinical trials demonstrated that nedaplatin should be administered at a dose of 30 to 35 mg/m^2^ every week in the concurrent Chemo-radiotherapy [8,9]. Yoshinage et al. conducted a dose-finding study and confirmed that the recommended dose was 35 mg/m^2^ every week [8]. Another phase I study of radiation therapy combined with nedaplatin showed that the optimal dose of weekly nedaplatin was 30 mg/m^2^. Nedaplatin could be given with minor adverse reactions and no delay in radiation therapy [9].

We conducted a single-centered phase II study to evaluate the tumor response rate, duration of response, survival time and adverse events of the concurrent chemo-radiotherapy with weekly nedaplatin in patients with advanced squamous cell carcinoma of the uterine cervix.

## Methods and materials

### Patients

Patients diagnosed with advanced squamous cell carcinoma of the uterine cervix were enrolled into this study according to the following criteria (Table 1). Written informed consent was obtained from all patients prior to enrollment. The Protocol was permitted by the Ethics Committee of the Cancer Institute and Hospital of the Chinese Academy of Medical Sciences.

**Table 1:** Inclusion criteria

(i)	Pathologically proven squamous cell carcinoma
(ii)	Clinical FIGO stage Ib and IIa2 with bulky tumor (>40 mm, assessed by magnetic resonance imaging) or Clinical FIGO stage IIb, IIIa, IIIb and IVa.
(iii)	No para-aortic lymph node swelling (≥10 mm) by abdominal computed tomography
(iv)	No prior radiation therapy for abdomen
(v)	Performance status (Eastern Cooperative Oncology Group): 0-2
(vi)	Age: 18 to 70 years old
(vii)	Adequate function of bone marrow, kidney and liver
	white blood cell count ≥ 2500 mm^3^
	neutrophil ≥ 1000 mm^3^
	hemoglobin ≥ 8.0 g/dl
	platelet count ≥ 75000 mm^3^
	creatinine ≤ 2.0 mg/dl
	GOT and GPT ≤ 2 times of the upper limit of normal at our institution
	T.Bil ≤ 2 times of the upper limit of normal at our institution)
(viii)	Written informed consent

### Treatment methods

#### Radiation therapy

##### External beam radiation therapy

The details of the external beam radiation therapy using 6MV X-ray were described below. The fraction dose was 1.8Gy, five fractions per week. Totaling 30.6Gy using the entire pelvic irradiation field without central shielding was followed by totaling 14.4Gy to 19.8Gy using the entire pelvic field with central shielding. Thus the total dose was 45.0Gy to 50.4Gy.

##### Intracavitary brachytherapy

Intracavitary brachytherapy, of which the fraction dose was 7 Gy to point A, was given once a week for a total of five to seven times. The total dose to point A was 35-49Gy.

##### Chemotherapy

Nedaplatin (30 mg/m^2^) was dissolved in 500 ml of 0.9% sodium chloride and infused intravenously over three hours. The first infusion was administered on the starting day of the external beam radiation therapy. The regimen was repeated weekly for six times.

The dosage of nedaplatin was decreased if grade 3 adverse events occurred; nedaplatin infusion was postponed if grade 4 adverse events occurred.

##### Response and toxicity evaluation

The incidence and severity of adverse events were evaluated according to the National Cancer Institute-Common Toxicity Criteria, version 3.0 [10].

The tumor response was defined following the guideline of the Response Evaluation Criteria in Solid Tumors (RECIST version 1.0) [11]. CR was defined as the complete disappearance of all measurable lesions for one month after completion of the treatment. PR was defined as a more than 30% reduction in measurable lesions. Progressive disease (PD) was defined as a more than 20% increase in measurable lesions or the appearance on one or more new lesions. Stable disease (SD) was defined as neither sufficient lesion shrinkage for PR, nor sufficient increase for PD. Patients were evaluated for response every four weeks by gynecological examination. In addition, radiological examinations were performed prior to the treatment and one month after completion of the treatment. The overall survival (OS) was defined as the time from the date of registration to death or the date of last contact. The progression free survival (PFS) was defined as the time from the date of registration to the date of last contact, disease progression, or death, whichever came first.

### Statistical design

The primary endpoint of this study was to assess the overall response rate and the adverse events. The secondary endpoint was to assess PFS and OS. The patients for the analysis of adverse events should receive at least one week nedaplatin. Meanwhile, the patients for the analysis of OS and PFS should complete the concurrent chemo-radiotherapy. The Kaplan-Meier method was used to estimate the overall and progression free survival time.

## Results

We enrolled thirty patients in this study from April 2010 to October 2010. Patient characteristics are presented in Table 2. The median age was 50.5 (ranging from 34 to 62). PS0 was 13 and PS1 was 17. Three patients were at the clinical stage IIa2, twenty were at stage IIb and seven were at stage IIIb.

**Table 2:** Patient Characteristics

Median Age, year-old (range)	50.5 (34–62)
Performance status, n (%)	
0	13
1	17
2	0
FIGO stage	
IIa2	3
IIb	20
IIIb	7
Histology	
Squamous cell carcinoma	30
Not Squamous cell carcinoma	0
Grade	
NC*	5
1	1
2	20
3	4

*NC: not confirmed.

Four patients experienced grade 2 to grade 4 myelosuppression after the administration of nedaplatin for one to three weeks and the administration of nedaplatin was discontinued because these patients did not recover within two weeks. One patient experienced grade 2 renal toxicity and the administration of nedaplatin was discontinued because the patient did not recover within two weeks. One patient experienced grade 2 of leukopenia and nausea, and voluntarily withdrew from the study. The above six patients were only evaluated for toxicity, not for response. Twenty-four patients who completed the treatment regimen (24 out of 30, 80%) were evaluated for response.

Acute hematological toxicities were observed, including grade 3 leukopenia in eight patients, grade 3 neutropenia in five patients, grade 3 anemia in two patients, grade 4 anemia in one patient, and grade 2 thrombocytopenia in six patients (Table 3).

**Table 3:** Hematological Toxicities (n = 30)

	**G0**	**G1**	**G2**	**G3**	**G4**
Leukopenia	1	6	15	8	0
Neutropenia	8	6	11	5	0
Anemia	18	7	2	2	1
Thrombocytopenia	15	8	7	0	0

Acute non-hematological toxicities included grade 2 liver disorder in one patient, grade 2 diarrhea in two patients, grade 2 nausea in two patients, and grade 2 renal toxicity in one patient. No patient experienced grade 3 or greater acute non-hematological toxicities.

Patients who completed the treatment regimen were evaluated for response rate one month after completion of the treatment. Twenty-three patients (95.8%) had CR and one (4.2%) had PR (Table 4).

**Table 4:** Objective Response (n = 24)

	**n**	**CR (%)**	**PR (%)**	**SD (%)**	**PD (%)**
All stages	24	23 (95.8)	1 (4.2)	0 (0)	0 (0)
Stage IIa2 - IIb	21	20 (95.2)	1 (4.8)	0 (0)	0 (0)
Stage IIIb	3	3 (3/3)	0 (0)	0 (0)	0 (0)

**Figure 1:**
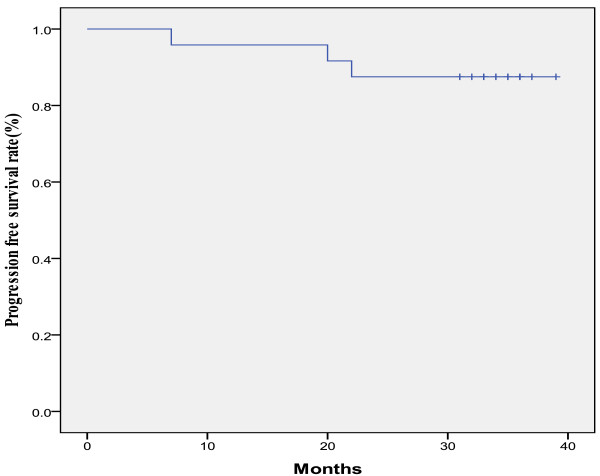
3-Year progression-free survival rate. The median follow-up duration was 36 months (range: 23–39).

**Figure 2:**
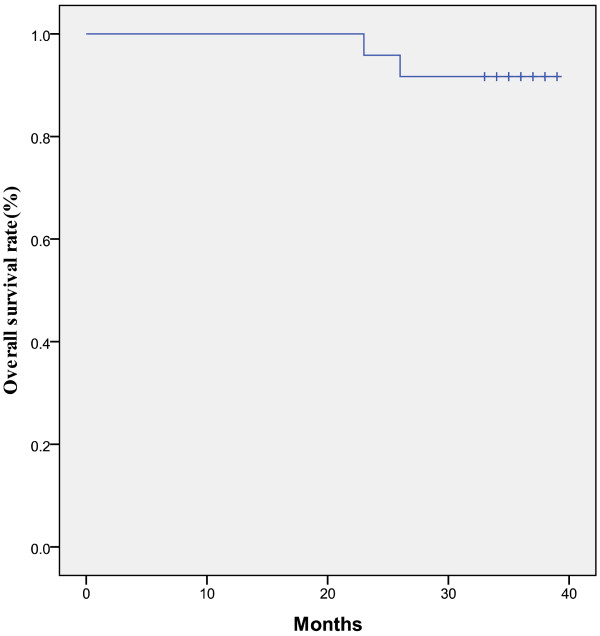
3-Year overall survival rate. The median follow-up duration was 36 months (range: 23–39).

The median follow-up duration was 36 months (range, 23–39), during which three patients relapsed after the treatment. Two patients with complete response relapsed (one patient relapsed inside the radiation field and the other in the lung). The only patient with partial response underwent three courses of chemotherapy shortly after completion of the treatment, and relapsed in the lung twenty months after the treatment. The 3-year PFS and OS rates were 87.5% and 91.7%, respectively (Figure 1, Figure 2).

The 3-year PFS rates were 100.0% (3/3), 88.9% and 66.7% (2/3) in patients with stage IIa, IIb and IIIb, respectively. Meanwhile, the 3-year OS rates were 100.0% (3/3), 88.9% and 100.0% (3/3) in patients at stage IIa, IIb and IIIb, respectively. These results were not significantly different (Table 5).

**Table 5:** 3-Year PFS and OS Rates

	**No. of patients**	**The 3-year PFS (%)**	**P-value**	**The 3-year OS (%)**	**P-value**
All patients	24	87.5	-	91.5	-
Stage					
IIa2	3	3/3		3/3	
IIb	18	88.9		88.9	
IIIb	3	2/3	0.360	3/3	0.710

## Discussion

The concurrent chemo-radiotherapy (CCRT) with cisplatin is currently the standard treatment for locally advanced uterine cervical carcinoma. However, the radiotherapy causes gastrointestinal adverse reactions, and cisplatin is associated with severe gastrointestinal adverse reactions. The CCRT thus inevitably aggravates gastrointestinal adverse reactions, which makes it more difficult for patients to tolerate. Cisplatin has severe renal toxicity as well. Therefore, many researchers tried to replace cisplatin with other agents as the radio-sensitizing agent in the CCRT for locally advanced uterine cervical carcinoma.

Nedaplatin (cis-diammine-glycoplatinum), a derivative of cisplatin, was developed in 1983 by Shionogi Pharmaceutical Company to provide a treatment with similar efficacy as cisplatin but less renal and gastrointestinal toxicities [12]. The preclinical evaluation of nedaplatin in cervical cancer demonstrated similar antitumor activity as cisplatin [13,14]. The incidence of nephrotoxicity was lower than that of cisplatin, due to the difference in drug distribution in the kidney. After the administration of the same dose, the amount of nedaplatin that accumulated in the rat kidney was approximately 40% of that of cisplatin, which explains why nedaplatin has less nephrotoxicity than cisplatin [15,16].

The radio-sensitizing properties of nedaplatin in the setting of CCRT for advanced uterine cervical carcinoma have been evaluated in two Phase I [8,9] studies, in which weekly 30–35 mg/m^2^ nedaplatin was recommended.

In a phase II study [17] of CCRT with nedaplatin for advanced uterine cervical carcinoma, the results showed that the response rate was 100% (80% for CR (8/10), and 20% for PR (2/10). In another phase II study [18] of CCRT with nedaplatin for advanced uterine cervical carcinoma, Yokoyama Y et al. reported that 40 of 45 enrolled patients completed the treatment. The response rate was 100% (90% had CR and 10% had PR). The median follow-up duration was 29 months (range: 8–52), the 3-year PFS and OS rates were 58.7% and 78.0% respectively. The response rate in this study was comparable to those in the two studies above. The 3-year PFS and OS rates in this study were higher than those in Yokoyama's report. The reason might be a lower proportion of patients at stage IIIb (12.5%).

In this study, a total of 30 patients were treated with radiotherapy plus concurrent chemotherapy. The dosage of nedaplatin chosen was weekly 30 mg/m^2^. Grade 3 leukopenia, neutropenia, anemia and thrombocytopenia were found in 26.7%, 16.7%, 6.7% and 0.0% of enrolled patients, respectively. Only one patient experienced grade 4 anemia (Table 3). There was no grade 3 or 4 non-hematological toxicity. 6 (20.0%) of the 30 enrolled patients withdrew from the trial due to myelosuppression, renal toxicity and nausea. The completion rate was 80% and the delayed duration was a maximum of 1 week. The complete response rate was 95.8% and all of the patients had a successful response. The 3-year PFS and OS rates were 87.5% and 91.5%, respectively. In addition, 80% of the enrolled patients completed the study with grade 4 hematological toxicities occurred in a few patients (3.3%), which indicated that weekly nedaplatin of 30 mg/m^2^ with concurrent radiotherapy was an effective and well-tolerated regimen for advanced uterine cervical carcinoma.

One of the limitations of this study was the small patient sample size. However, the results are sufficient to warrant further research. A randomized phase III study of this regimen were needed to validate whether nedaplatin in concurrent chemo-radiotherapy is a better choice than cisplatin with respect to the survival of patients with advanced squamous cell carcinoma of the uterine cervix.

## Conclusion

This phase II study suggested that concurrent chemo-radiotherapy with weekly nedaplatin was effective and safe for the treatment of advanced squamous cell carcinoma of the uterine cervix.

## Abbreviations

CCRT: The concurrent chemo-radiotherapy; CR: Complete response; NCI: National Cancer Institute; OS: The overall survival; PD: Progressive disease; PFS: The progression free survival; PR: Partial response; SD: Stable disease.

## Competing interests

The authors declare that they have no competing interests.

## Authors’ contributions

GY and LW drafted the manuscript and performed the data analysis. GY, NL and JA performed the data collection. GY, LW and MH participated in the study design and coordination. GY, LW and MH discussed the outcome during the study. All authors read and approved the final manuscript.
